# Stereotactic body radiotherapy in patients with lung tumors composed of mainly ground-glass opacity

**DOI:** 10.1093/jrr/rraa015

**Published:** 2020-03-26

**Authors:** Hiroshi Onishi, Yoshiyuki Shioyama, Yasuo Matsumoto, Yuta Shibamoto, Akifumi Miyakawa, Gen Suzuki, Yasumasa Nishimura, Ryohei Sasaki, Daisuke Miyawaki, Kengo Kuriyama, Takafumi Komiyama, Kan Marino, Shinichi Aoki, Ryo Saito, Masayuki Araya, Yoshiyasu Maehata, Hotaka Nonaka, Licht Tominaga, Masahide Saito, Naoki Sano, Shogo Yamada

**Affiliations:** 1 Department of Radiology, School of Medicine, University of Yamanashi, Japan; 2 Ion Beam Therapy Center, SAGA-HIMAT Foundation, Japan; 3 Department of Radiation Oncology, Niigata Cancer Center Hospital, Japan; 4 Department of Radiology, School of Medicine, Nagoya City University, Japan; 5 Department of Radiation Oncology, School of Medicine, Kurume University, Japan; 6 Department of Radiation Oncology, Kindai University Faculty of Medicine, Japan; 7 Department of Radiation Oncology, School of Medicine, Kobe University, Japan; 8 Department of Radiation Oncology, School of Medicine, Tohoku University, Japan

**Keywords:** stereotactic body radiation therapy (SBRT), lung cancer, ground glass nodule (GGN), ground glass opacity (GGO), stage I

## Abstract

We retrospectively reviewed the effect of stereotactic body radiation therapy (SBRT) in patients with stage I lung cancer whose lung tumor showed a nodular appearance of ground glass opacity, so-called ground glass nodule (GGN). A total of 84 patients (42 men, 42 women; mean age, 75 years) with stage I lung cancer with GGN accompanying a solid component <50% in diameter of the tumor and no metastases were studied. Concerning histology, 32 tumors were adenocarcinoma, 1 was squamous cell carcinoma, 2 were unclassified carcinoma and 49 cases were histology-unproven but increased in size or had a positive finding in ^18^F-FDG positron emission tomography (PET) examination. The median tumor size was 20 mm (range, 10–41 mm). All of the patients were treated with SBRT, and the total prescribed dose at the isocenter ranged between 48 Gy in four fractions and 84 Gy in ten fractions. Median follow-up duration was 33 months. No patient had local failure nor regional lymph node failure. The 3-year rate of distant failure was 2.6%. Two patients who experienced distant metastases had a past surgical history of initial lung cancer before SBRT. The rates of cause-specific and overall survival at 3 years were 98.2 and 94.6%, respectively. Treatment-related adverse events of ≥grade 4 were not reported. Although more cases and longer follow-ups are mandatory, SBRT may be one of the radical treatment options for patients with GGN.

## INTRODUCTION

Recent routine examinations using computed tomography (CT) in various situations in clinical practice has increased the frequency of discovering lung tumors showing an appearance of ground glass opacity (GGO). The optimum treatment strategy for such a lung tumor, so-called ground glass nodule (GGN) is unclear, but surgery has been generally performed as a general treatment for GGN, and its prognosis is better than that of solid-type tumors [1]. Hypofractionated stereotactic body radiation therapy (SBRT) was introduced by Blomgren *et al*. [2] and Uematsu *et al*. [3], and it requires some advanced programing, including image-guided precise localization of the tumor during the treatment process, avoiding tumor motion due mainly to respiration, and highly conformal dose concentration to the tumor with a steep dose gradient to minimize dose to surrounding healthy tissue. SBRT is commonly performed in patients with small-sized primary or oligometastatic lung tumors as a radical and minimally invasive treatment in a shorter course requiring fewer visits to the clinic than conventional radiation therapy. Recently, promising outcomes of SBRT for medically operable patients with stage I non-small cell lung cancer (NSCLC) have been reported [4, 5], but there are few reports presenting a specified outcome in patients with GGN treated with SBRT. Tsurugai *et al*. [6] reported that the ratio of consolidation to total tumor in diameter (CTR) predicted the outcomes of patients who received SBRT for NSCLC, and CTR was the only significant predictor of disease-free survival, but the patient number of the CTR <0.5 group was small (*n* = 31). Therefore, the purpose of this study was to investigate outcomes following SBRT in patients with clinical stage I lung cancer with a GGN with CTR <0.5 in our multicenter database.

## MATERIALS AND METHODS

**Fig. 1.**
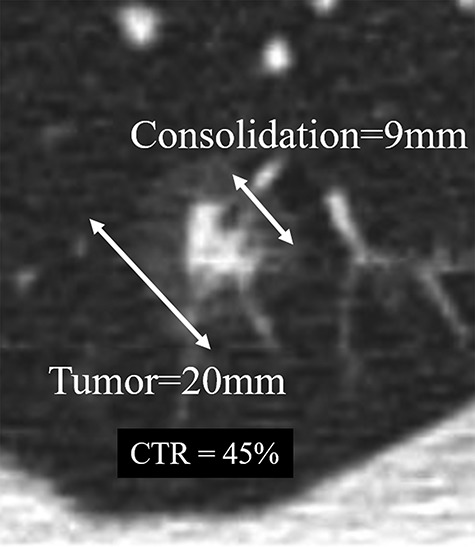
An example of calculation of the ratio of consolidation to total tumor containing GGO in diameter (CTR). GGO was defined as hazy opacity that does not obscure underlying bronchial structures or pulmonary vessels. Consolidation was defined as dense opacity that obscures underlying bronchial structures or pulmonary vessels. In this case, CTR was 9/20 = 45%.

We used a retrospective study design. ‘GGO’ and ‘consolidation’ were defined as hazy opacity that did not obscure underlying bronchial structures or pulmonary vessels and dense opacity that obscured underlying bronchial structures or pulmonary vessels, respectively, on images of high-resolution CT. High-resolution CT scanning was performed with slice thickness <3 mm and the high-resolution image was reconstructed in 5–10 min and displayed with lung field conditions (window width: 1200 Hounsfield unit (HU) to ~1500 HU, window level: 500 HU~ to 700 HU). An example of calculation of the ratio of consolidation to the total GGN in diameter (CTR) is shown in Fig. 1. We reviewed 84 patients who had GGN with its CTR <50% without any metastatic findings and who were treated with SBRT in the seven institutions that were included in the Japanese multi-institutional study group of SBRT. Medical operability was judged according to each institution’s criteria. Local progression after SBRT was judged comprehensively by CT, PET (positron emission tomography) or biopsy. Table 1 summarizes the clinical characteristics of patients and dose-fractionation of SBRT. SBRT was performed because of pathological proof, growth of the tumor or positive finding in ^18^F-FDG PET examination. Histological diagnosis was proved in 40 patients and most of them are adenocarcinomas. SBRT was performed using non-coplanar multiple static ports or dynamic arcs and respiratory motion control, as required. Clinical target volume was equal to the gross tumor volume, and internal margin (IM) and generally 5-mm setup margin were added to create the planning target volume. The IM was calculated according to the respiratory motion management and image-guidance technique of each of the institutions. The dose constraints of the organs at risk, such as lung, spinal cord, esophagus, pulmonary artery, gastrointestine and trachobronchus were set to avoid serious complications in accordance with the protocol of the study JCOG0403 [7] using a linear-quadratic model when the total fraction number was different from that of JCOG0403. Patients were fixed to avoid an inter-treatment set-up motion error of more than 5 mm in any direction. The algorithm for dose calculation was pencil beam or similar in 78 patients and superposition or similar in 17 patients. Total prescribed dose at the isocenter ranged between 44 Gy in four fractions and 84 Gy in ten fractions. The biological effective dose was calculated using a linear quadratic model (at α/β ratio = 10 Gy) distributed from 92.4 Gy to 154.6 Gy. During the follow-up, generally regular CT was taken at 3–6 months intervals, and biopsy was tried when local recurrence was suspected. The prognosis regarding local recurrence, regional lymph node metastases, distant metastases, survival status and cause of death were examined. Survival and metastases rates were calculated using the Kaplan–Meier method and confidence interval (CI) was calculated with Greenwood’s formula. Statistical difference was calculated with a log-rank test. Differences were judged as statistically significant when *P* < 0.05. All statistical analyses were performed using Statview software (SAS, Cary, NC, USA).

**Table 1:** Patients and tumor characteristics

Total number of patients	84
Gender	Male: 42
	Female: 42
Age	50–92 (median 75) years
Histology	Aenocarcinoma: 32
	Squamous cell cancer: 1
	Unspecified non-small cell lung cancer: 2
	Unproven: 49
Operability	Operable: 39
	Inoperable: 45
History of surgery for lung cancer	Yes: 8
	No: 76
Tumor size	10–41 mm (median 22 mm, ≥20 mm: 49 cases)
T stage (7th UICC)	T1a:T1b:T2a = 39:32:13
CTR	0–50% (median 26%, ≥25%: 53 cases)
Total dose and fraction (biological effective dose^a^)	44 Gy/4 fraction (BED 92.4 Gy): 1
	48 Gy/4 fraction (BED 105.6 Gy): 54
	50 Gy/4 fraction (BED 112.5 Gy): 2
	52 Gy/4 fraction (BED 119.6 Gy): 21
	60 Gy/8 fraction: 3
	70 Gy/10 fraction: 3

^a^BED, biological effective dose (α/β = 10 Gy).

This study was approved by the Institutional Review Board (approval number 961) and opted-out in each institution.

## RESULTS

**Fig. 2.**
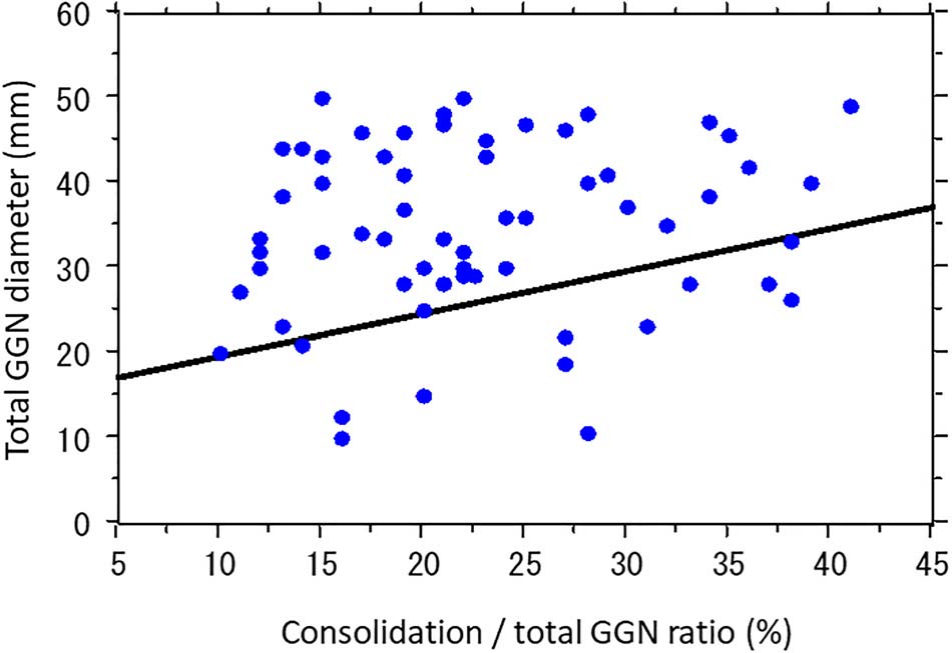
The relationship between tumor size and consolidation/GGN ratio. The correlation coefficient and *P*-value of the regression coefficient between these two factors were 0.22 and 0.043, respectively.

The relationship between tumor size and CTR is shown in Fig. 2. The correlation coefficient and *P*-value of the regression coefficient between these two factors were 0.22 and 0.043, respectively. Median follow-up duration was 33 (range 7–78) months. No patients had a local failure in the planning target volume or regional lymph node metastases. A summary of the results is shown in Table 2. Only two patients experienced distant metastases and the 2-year rate of distant failure was 4.0% (95% CI: 0.0–8.9%). Both of the patients had a history of lung cancer treated with surgery. The size and CTR of the tumor in one patient who had brain metastases were 11 mm and 0%, respectively, but the patient had a history of surgery for T2N1 lung cancer 34 months before SBRT. The size and CTR of the tumor in the other patient who had bone metastases were 22 mm and 0%, respectively, and the patient had a history of surgery for T2N0 lung cancer 5 months before SBRT. The 2-year rate of distant failure of the patient subgroup with a history of lung cancer was 25.0% (95% CI: 0.0–55.0%).

**Table 2:** Summary of results

	Median follow-up: 33 months
Local progression	0/84 (0.0%)
Regional lymph node metastases	0/84 (0.0%)
Distant metastases	2/84 (2.4%)
2-Year overall survival rate	98.7% (95% CI: 96.3–101.2%)
2-Year disease-free survival	96.0% (95% CI: 91.5–100.4%)
2-Year lung cancer-specific survival rate	100.0% (95% CI: 100.0–100.0%)

**Fig. 3.**
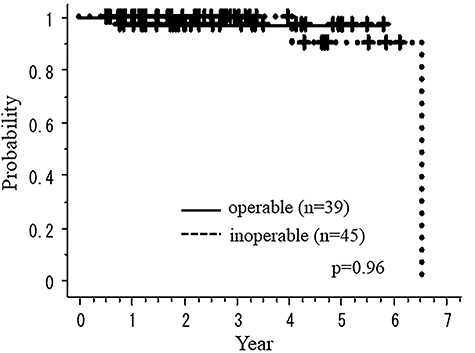
Overall survival rate according to medical operability.

**Fig. 4.**
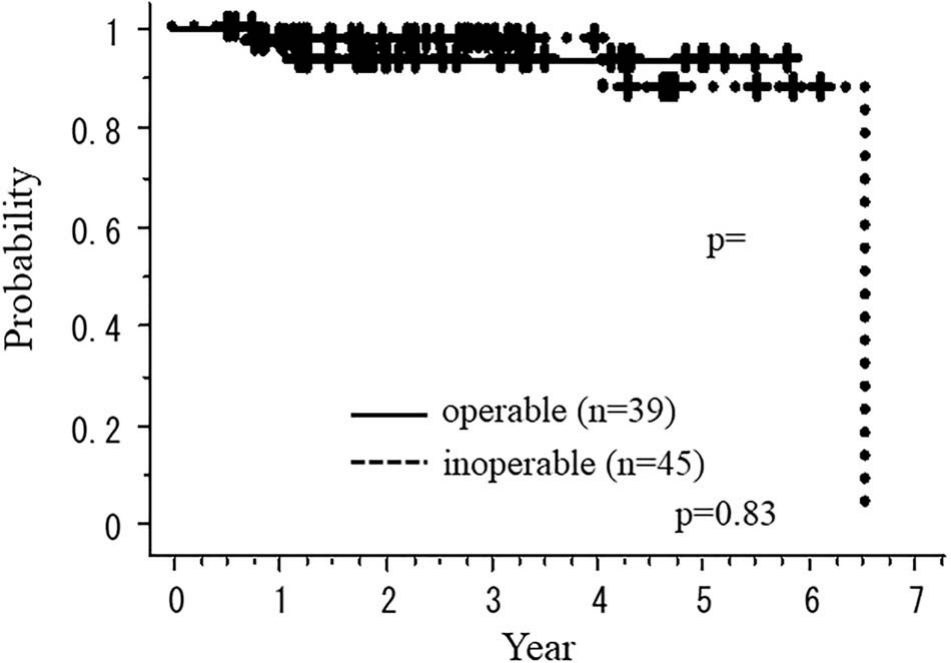
Recurrence-free survival rate according to medical operability.

**Fig. 5.**
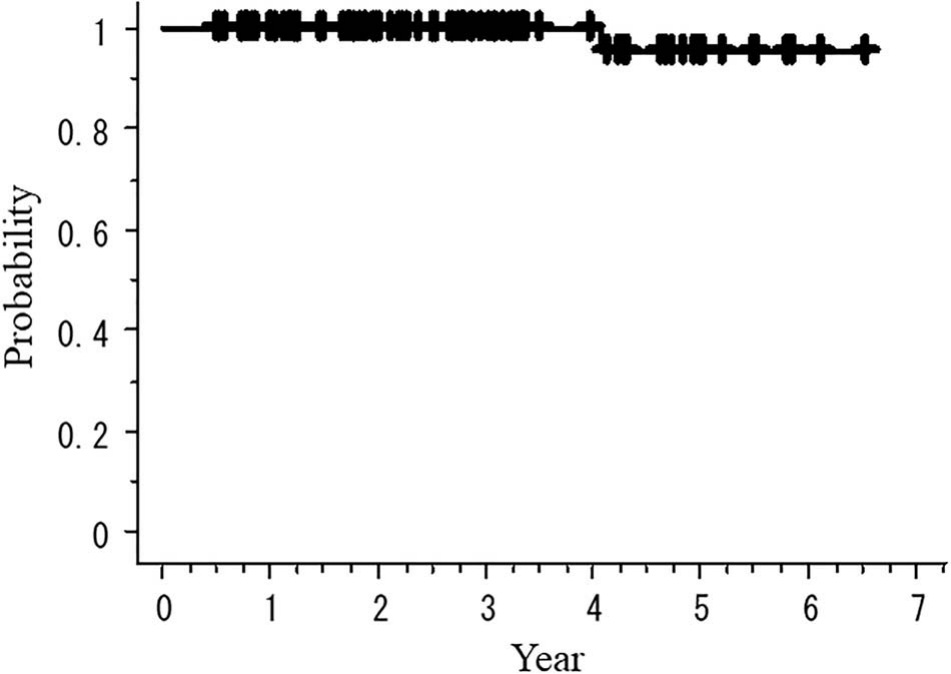
Lung cancer-specific survival rate.

Figs 3 and 4 show the overall survival rate (OS) and recurrence-free survival rate (RFS) according to medical operability, respectively. Figure 5 shows cause-specific survival rate (CSS). The OS of total, operable and inoperable patients at 3 years were 98.7 (95% CI: 96.3–101.2%), 97.1 (95% CI: 91.6–102.7%) and 100.0% (95% CI: 100.0–100.0%), respectively. There were no statistical differences in OS and RFS between operable and inoperable subgroups. The 3-year RFS and CSS rates of all patients were 96.0 (95% CI: 91.5–100.4%) and 100.0% 91.5–100.4, respectively. The 3-year OS of the histology-proven subgroup was 100.0% (95% CI: 91.5–100.4%). There were no local recurrences nor metastases in the cases whose tumors were initially GGN with follow-up >5 years.

The treatment-related adverse events were almost mild and grade 4 adverse events were not observed.

## DISCUSSION

The popularity of CT screening resulted in an increase of detection of GGN. Noguchi *et al.* reported the relationships between thin-sliced CT findings with a GGO component of lung adenocarcinoma with pathologic classification and prognostic factor [8]. In their results, the frequencies of lymph node metastases and vessel invasion in tumors with C/G ratio <50% were significantly lower and the survival rate was better than in the others. Therefore, we selected the patients with GGN for which CTR was <50%.

In this study, local recurrence and regional lymph node recurrence were not found in all patients, but the follow-up duration was too short to validate the true efficacy and necessity of SBRT for patients with small GGN with a CTR <50%. Regarding distant metastases, two cases showed bone or brain metastasis. In both cases, the patient had a history of surgical resection for previous solid-type lung cancer; thus it might be considered that the origin of the metastases was a previous lung cancer site, not tumors treated with SBRT. Hiramatsu *et al*. reported that a history of lung cancer was a risk factor for GGN growth, and therefore this should be kept in mind during follow-up [9]. Haro *et al*. reported that newly emerging GGN on postoperative CT had significantly more malignant radiological findings than other preoperatively existing GGN [10].

Regarding survival, the OS of our results was better than that reported in the JCOG0403 study [7], which included any type of tumor regardless of CTR. Tsurugai *et al*. also demonstrated that the 3-year OS for the CTR <0.5 group was 87.5% [6]. However, these results must be compared with that of surgery. Asamura *et al*. reported that the 5-year OS for 121 patients with T1 tumors with CTR <0.5 was 96.7% with a 7.1 years median follow-up period [11].

We found a strange but interesting result in our study that OS of medically operable and inoperable groups were almost the same, although the former was generally higher than the latter in several reports [3, 4, 7]. The reason is unclear, but it may be due to etiological differences in the groups of GGN and solid tumors, such as smoking history [12]. According to the prospective study for pathological finding of peripheral lung tumors with GGO appearance, radiological noninvasive peripheral lung adenocarcinoma could be defined as an adenocarcinoma ≤2.0 cm with ≤0.25 consolidation [13]. In our study, there was no recurrence when excluding postoperative recurrent cases, although more than half of the patients had tumors >2 cm in diameter or 25% CTR.

There were some limitations and arguments with regard to our study. First, it was a retrospective study with a small number of patients and the follow-up period is too short (median 33 months) for such indolent disease, as the natural history of such tumors showing the appearance of mainly GGO is often measured in years even without treatment. Therefore, a longer follow-up period within a prospective setting is needed to determine whether SBRT is indicated in such cases. However, the result could be meaningful, at least for elderly patients whose residual life time is considered to be not so long, because more than half of the cases were >2 cm in size or 25% CTR. Second, the method for prescription and its calculation algorithm differed in the institutions, therefore a true evaluation with regard to the dose was impossible. Third, it is sometimes difficult to differentiate a local recurrence from post-SBRT inflammatory change [14, 15]. Thus, the true local control rate is unknown and it would be difficult to judge when to add a salvage therapy for local recurrence after SBRT. Finally, there is an argument that it is questionable to perform SBRT for GGN, particularly in cases without pathological confirmation, and immediate treatment leads to an overtreatment for such tumors. When GGNs are detected, follow-up CT scans are currently recommended by the Fleischner society [16]. However, it was reported that the possibility of invasive adenocarcinoma got higher when the CTR was >0.25 and the diameter was >2 cm in a GGN [11] Therefore, it is commonly recommended that surgical resection should be considered in the cases with GGNs with CTR >0.25 or diameter >2 cm. Actually in these patients, two local treatments of wide wedge resection and segmentectomy are being compared in an ongoing prospective randomized trial conducted by the Japan Clinical Oncology Group [17]. Thereafter, SBRT could be considered as one of locally radical treatment strategies.

It has also been argued that pathological examination must be performed in order to acquire molecular-level information such as epidermal growth factor receptor (EGFR) or echinoderm microtubule-associated protein-like 4 (EML4)-anaplastic lymphoma kinase (ALK) mutation status and programmed cell death-1 (PD-L1) in order to consider SBRT as an adjuvant or salvage therapy. However, most recurrences of these types of tumors would appear after some years; thus, the molecular or immunological information may change and it must be confirmed again by re-biopsy or resection when the tumor recurs. Consequently, as SBRT is generally less-invasive than surgery [5], the SBRT strategy would be permitted in cases without pathohistological examination.

## CONCLUSION

SBRT for patients with clinical or pathological stage I lung cancer with lung tumor of with CTR <50%, so-called GGN, resulted in a promising survival rate with few local progressions, lymph node metastases and distant metastases. Although more cases and longer follow-up are mandatory, SBRT would be one of the radical treatment options for patients with clinical or pathological stage I lung cancer with the lung tumor representing GGN and its CTR <50%.
